# Construction and validation of an in-hospital transport simulation scenario

**DOI:** 10.31744/einstein_journal/2021AO5868

**Published:** 2021-09-09

**Authors:** Ellen Reis Santana, Luiz Humberto Vieri Piacezzi, Maria Carolina Barbosa Teixeira Lopes, Ruth Ester Assayag Batista, Cássia Regina Vancini-Campanharo, Aécio Flávio Teixeira de Góis

**Affiliations:** 1 Escola Paulista de Medicina Universidade Federal de São Paulo São PauloSP Brazil Escola Paulista de Medicina, Universidade Federal de São Paulo, São Paulo, SP, Brazil.; 2 Escola Paulista de Enfermagem Universidade Federal de São Paulo São PauloSP Brazil Escola Paulista de Enfermagem, Universidade Federal de São Paulo, São Paulo, SP, Brazil.

**Keywords:** Simulation technique, Education, nursing, Patient transfer

## Abstract

**Objective:**

To build and validate the content of a clinical simulation scenario for teaching in-hospital transport of critically ill patients.

**Methods:**

A descriptive study of construction and validation of a clinical simulation scenario for teaching in-hospital transport. A scenario based on the literature was built, followed by validation, using the Delphi technique, by five specialists, with an agreement of 80%. The experts were selected through snowball sampling. An instrument was developed containing 26 topics to be assessed for relevance, clarity, objectivity, feasibility, current content, vocabulary, and a field for observations.

**Results:**

Two rounds were carried out by the specialists to reach a consensus of 80%. According to the experts´ recommendation, the initial scenario was written more clearly and objectively, and divided into two parts: patient preparation and patient transport.

**Conclusion:**

In this study, the construction and validation of the scenario for teaching in-hospital transport were successfully performed. It may be applied in several services to evaluate the training of nursing undergraduate students, as well as for the professional improvement of those who work in the in-hospital transport service.

## INTRODUCTION

Clinical simulation is an educational technique that enables performing scenarios to reproduce a real-life situation, promoting patient-centered teaching and learning, and allowing the student to experience the representation of an event from a clinical perspective, addressing a wide variety of situations with different degrees of complexity, and the development of multiple skills and decision-making.^(1-4)^

With the growing use of clinical simulation as an active methodology in health sciences teaching, the need arose for the establishment of a method for the construction of scenarios, since the absence of a script may compromise the efficacy of the activity.^(4,5)^

Thus, the construction of the clinical simulation scenario requires the fulfillment of all steps, including literature search, determination of objectives, construction of the clinical situation, preparation of the content, facilitator, and venue, and planning of the materials and resources necessary for the development of the activity.^(1,3)^

The description of the scenario should not be based solely on clinical experiences, since these may not present all pieces of information required to achieve the learning objectives. In this context, the validation process through consensus among experts contributes to the clinical simulation being effective, and including all items for the appropriate development of the skills to be acquired.^(6-8)^

When considering the high rate of adverse events (drop in saturation, blood pressure and heart rate modifications, technical failures of the equipment used to ensure patient safety, reduction of avoidable harm that threatens the patient’s life during transportation) in the in-hospital transport of critically ill patients, it is vital to reinforce the importance of good practices, carrying out simulated training based on updated guidelines. In these, clinical reasoning, optimized evaluation, and definition of risks and benefits related to transport are analyzed.^(9-13)^

Thus, clinical simulation for teaching students to perform in-hospital transport of critically-ill patients grows in importance, since it allows applying a high-fidelity simulated training, replicating the challenges, possible instabilities, and clinical complications faced by health professionals in everyday life, and developing skills for constant complex decision-making, prevention of possible adverse events, and strategies that meet the therapeutic needs of critically ill patients in a completely safe and controlled environment, and above all, free of risks to professionals/patients.^(4,8-11)^

Bearing in mind that the implementation of pedagogical activity is relevant for learning, with the necessary association of theory and practice, the use of strategies that contribute to a better understanding of the phenomena is essential for the training of health professionals, and thus, patient safety is prioritized.

## OBJECTIVE

To build and validate the content of a clinical simulation scenario for teaching in-hospital transport of critically ill patients.

## METHODS

This is a descriptive study of construction and validation of a scenario in simulated clinical activity on in-hospital transport of critically ill patients. The study was developed during the period from August 2017 to March 2018, in two stages: in the first stage, the scenario about in-hospital transport of critically ill patients was built based on the literature. In the second stage, the scenario was validated using the Delphi technique, which allows consensus to be reached among a group of experts in the field of knowledge about a given phenomenon. In this case, about simulated teaching in healthcare. There is no consensus in literature as to an ideal number of experts; a minimum of five is suggested as sufficient for agreement control.^(6,7)^Considering the value recommended in the literature is higher than 70%, we used five experts in this study, and the level of agreement considered among them for scenario validation was 80%.^(14)^

The experts were selected through snowball sampling, and the selection criteria were to have a Master’s and/or Doctoral degree in health or education, and a minimum of 2 years of experience in teaching health simulators at a higher education organization.^(15)^ The specialists were e-mailed an invitation letter along with the Informed Consent Form. After their acceptance, they answered a questionnaire with information for professional characterization and another to evaluate the scenario.

The first version of the scenario was developed with the following topics: description of the theme, target audience, requirements to participate in the activity, number of participants, duration of the scene and debriefing, learning objectives, accuracy, complexity, materials needed, guidelines for preparing the scenario, and actions expected at each moment. As some topics had more than one item, the structured instrument had 26 items to be content validation as to pertinence, clarity, objectivity, feasibility, current content, and vocabulary. There was also an open field for comments, in case the examiner thought it necessary to make them.^(16)^

The study was approved by the Research Ethics Committee of the *Universidade Federal de São Paulo* (CAAE: 80661017.7.0000.5505, opinion 2.503.340).

## RESULTS

The specialists selected to validate the scenario were mostly female (80%), with a mean age of 43 years (±7.33). Regarding education, all of them were educated in the health area, and 80% had a PhD degree. Regarding experience with clinical simulation, all had participated in the training course for instructor in simulation, symposia, and conferences in the field, with a mean experience of 9.6 years (±6.58), and 20% reported working at public organizations, 60% in private organizations, and 20% in both. Two rounds of expert evaluation were performed to obtain at least 80% agreement. In the first, agreement was obtained in 24 items and disagreement in two. This was the description of the clinical case in which consensus among judges was 40%, and the item referring to the time of the activity, with 60% consensus (Table 1).

**Table 1:** Disagreement among experts in the first round

Disagreeing items	Percentage of disagreement					
	Pertinence	Clarity	Objectivity	Feasibility	Current content	Vocabulary
Description of the clinical case (scenario)	100	40	40	100	80	80
Time limit of the activity (preparation for transport)	80	80	60	60	100	100

According to the experts’ recommendation, the case was written more clearly and objectively, and the initial scenario was divided into two parts: patient preparation and patient transport. Each scene was 10 minute-long, with a 20 minute-debriefing. Table 2 shows the final version of the scenario. This time, agreement of at least 80% was obtained for all items evaluated.

**Table 2:** Final version of a clinical simulation scenario for in-hospital transport of the critically ill patient

Scenario: simulation of in-hospital transport of the critically ill patient					
Theme: in-hospital transport of the critically ill patient					
Target audience: 4^th^ year undergraduate nursing students					
Requirements:	To have taken the theoretical class on in-hospital transport of the critically ill patient To know the material resources necessary to perform the transport To know the main adverse events that occur during transport and how to intervene				
Number of students: 4					
Scenario 1: preparation for in-hospital transport of the critically ill patient					
Duration of simulated activity: 10 minutes		Debriefing: 20 minutes			
Scenario 2: in-hospital transport of the critically ill patient					
Duration of simulated activity: 10 minutes		Debriefing: 20 minutes			
At the end of this training, the student should be able to:					
General objective:	Perform in-hospital transport, aiming to ensure safety and prevent/reduce the occurrence of adverse events				
Specific objectives:	Orient the patient and the companion about the performance and purpose of the in-hospital transport Assess the patient’s clinical conditions and documentation for in-hospital transport Plan the in-hospital transport Assemble the appropriate team and the necessary equipment for the in-hospital transport Register the transport, complications, and respective interventions, if any, in the medical record				
In-hospital transport					
In-hospital transport is defined as the temporary or definitive transfer of patients by health professionals within the hospital environment, ensuring their integrity and safety^(1)^ Critically ill patients require interventions that often cannot be performed in bed, requiring transportation for diagnostic or therapeutic procedures. Adverse events are frequent during in-hospital transport of critically ill patients, and may lead to respiratory and hemodynamic instability, which often result from lack of knowledge of professionals, communication failures between the team, and equipment used during transport. Thus, safety during in-hospital transport is extremely important and can be achieved with teaching and simulated training of good practices, aiming at clinical reasoning for accurate assessment of risks and benefits related to transport, and the development of technical skills for its realization, to ensure patient safety and reduce the occurrence of preventable damage during the route.^(2-5)^ The nursing student needs to understand the importance of the correct identification of the patient to be transported, as well as the purpose of the transport, planning, and carrying out the transport, preventing or reducing the occurrence of adverse events. Thus, it is fundamental that the nursing student develops the following skills: communication, organization, critical thinking, dexterity, safety, and leadership. These actions have the purpose of preventing and reducing adverse events during transport					
Scenario 1: preparation for in-hospital transport of the critically ill patient					
Briefing: before the beginning of the activity, give a presentation of the case, the environment, and the resources available for care					
Case summary: Mr. J.S., age 42 years, was a victim of a fall from approximately 5m height, 48 hours ago, and will be referred for examination since he presented with a decreased level of consciousness. A computed tomography scan was ordered and the Diagnostic Center is already waiting. Mr. J.S. is sedated, with a clean and dry external dressing on the head, closed nasoenteric tube, maintaining orotracheal intubation on mechanical ventilation - volume controlled mode, 50% fraction of inspired oxygen (FiO2), central venous catheter in the right subclavian vein receiving propofol 15mL/L, and an indwelling urinary catheter with a closed drainage system. There is no description of allergies in the medical record. Vital signs: blood pressure (BP): 120x60mmHg; heart rate (HR): 79bpm; respiratory rate (RR): 14rpm; oxygen saturation: 96% and RASS: -5					
Preparation of the high-fidelity set and manikin					
The patient/manikin must be in bed with raised head, rails up, and wheels locked The patient/manikin should be in the following conditions/with the following devices: closed nasoenteric tube in right nostril; orotracheal cannula coupled to mechanical ventilator, central venous catheter in right subclavian vein coupled to an infusion pump system; identification bracelet on right upper limb; and indwelling urinary catheter coupled to a closed drainage system					
Material needed for the set					
Patient/manikin Identification bracelet Bed Procedure gloves Tracheal cannula no. 7.0 Fixation for tracheal cannula Complete mechanical transport ventilator Oxygen cylinder Complete transport monitor/defibrillator Electrodes Crepe bandage Crepe tape Nasoenteric tube Indwelling urinary catheter Closed collector for indwelling urinary catheter Saline support attached to the bed Bag of saline solution 0.9% Infusion pump set Infusion pump Electrodes for cardiac monitoring Central venous catheter Request for test Medical prescription Control sheet Medical chart		Carrying case containing: - Procedure gloves - surgical gloves - Bag-valve-mask device - Laryngoscope kit with backup battery - Tracheal cannula (several sizes - adult) - Guidewire - Cannula fixation material - Laryngeal mask (several sizes - adult) - Peripheral venous catheter (several sizes - adult) - Spigot - Hypoallergenic tape for bandages - Syringe (several sizes) - Needle (various sizes) - Infusion set (macro drops) - Infusion set (for pump) - Saline solution 0.9% (ampoules and bag) - 70% alcohol - Absorbent cotton Emergency medications: - Hypertonic glucose - Epinephrin - Amiodarone - Atropine - Etomidate - Fentanyl - Midazolam - Succinylcholine - Diazepam			
Preparation for transport					
Expected actions:		Guidelines/parameters provided:			
Check the test order and the complete name on the patient’s wristband		( ) Order identification matches the patient’s identification			
Instruct the accompanying person on the performance and purpose of the transport		( ) The student instructs the accompanying person on the need to transport the patient to the Diagnostic Center for a computed tomography scan of the skull			
Get the medical chart		( ) The chart is on the counter			
Check the patient’s chart for allergy history and preparation for the test		( ) The student identifies the patient has no history of allergies and maintains fasting			
Assess the patient’s level of consciousness/sedation		( ) Student identifies the patient is receiving the sedative propofol ( ) Student applies the Richmond Agitation-Sedation Scale (RASS) and identifies RASS=-5			
Assess the patient’s ventilatory/respiratory status		( ) The student checks the positioning of the tracheal tube through pulmonary auscultation, which is present bilaterally ( ) The student checks the fixation of the tracheal cannula, which is adequate The student checks the ventilatory parameters: ( ) Volume controlled mode ( ) Tidal volume=500mL ( ) FiO2=50% ( ) Respiratory rate=14rpm ( ) PEEP=5mmHg ( ) Student checks oxygen saturation, which is 96%.			
Assess heart rate and blood pressure		( ) The student assesses the heart rate, which is 79bpm ( ) The student assesses the blood pressure, which is 120/60mmHg			
Check the prescription to see which infusions the patient is receiving and for how long		( ) The student identifies that the patient is receiving propofol by infusion pump at 15mL/hour and the volume of solution (200mL) is sufficient for transportation to the intensive care unit			
Adapt the necessary equipment for transportation		The student must adapt the: ( ) Transport ventilator ( ) Oxygen cylinder ( ) Transport monitor/defibrillator ( ) Saline drip stand ( ) Continuous infusion pump			
Request the team that will do the transportation		( ) The student requests the team, which is available			
Shift handover/effective communication		The team that prepared the patient communicated to the team that will perform the in-hospital transport, about: ( ) Clinical conditions of the patient ( ) Test that will be performed ( ) Preparation for the test			
The scenario must be interrupted when: at the end of the proposed activity or reaching the 10-minute time limit					
Scenario 2: in-hospital transport of the critically ill patient					
Expected actions:		Orientations/parameters given:			
Shift handover/effective communication		The team that will perform the in-hospital transport properly did handover and confirmed: ( ) Clinical conditions of the patient ( ) Tests that will be performed ( ) Preparation for the test			
Assess the distance to be traveled, possible obstacles, and time to be spent at the destination		( ) Students discuss among themselves the distance to be traveled, whether there will be any obstacles that might hinder transportation, and the time allotted			
Anticipate possible complications that may occur with the patient during the journey		The student discusses possible complications with the transport team: ( ) Displacement of the tracheal/extubation ( ) Transport ventilator failure ( ) Drop in saturation ( ) End of oxygen supply from the cylinder ( ) Hemodynamic instability ( ) Displacement/loss of central venous access ( ) Running out of propofol solution ( ) Failure of transport monitor/defibrillator ( ) Nasoenteric tube displacement/loss ( ) Displacement/loss of indwelling urinary catheter			
Carry out communication between the unit of origin and the unit receiving the patient		( ) The student communicates with the Diagnostic Center, informing them the patient is being referred for testing			
Keep the rails raised to ensure the patient’s physical integrity		( ) The student raises and keeps the rails up all the way			
Transport the patient to the Diagnostic Center		( ) Determine the team that will be responsible for transporting the patient			
		Adverse event		Expected action	
Intervene during transport if the adverse event occurs		( ) Tracheal tube displacement/extubation		( ) Maintain ventilation with bag-valve-mask device ( ) Assist the physician in repositioning the tracheal cannula as soon as possible	
		The patient will present with a drop in saturation:			
		( ) Transport ventilator failure			( ) Maintain ventilation with bag-valve-mask device ( ) Correct failure, if possible ( ) Replace transport ventilator
		The ventilator will sound the alarm and display the message “LOW VOLUME”:			
		( ) End of oxygen supply from the cylinder			( ) Replace oxygen cylinder
		( ) Tracheal cannula cuff deflated			( ) Listen for air escaping from the patient’s mouth ( ) Auscultate the patient’s cervical region and confirm the passage of air through the site ( ) Check the low cuff tension ( ) Inflate the cuff until the air leak is corrected
		Displacement/loss of central venous access:			
		( ) The student identifies that access is externalized ( ) Yes ( ) No			( ) Puncture new peripheral venous access
		The pump sounds the alarm with the message “KVO”:			
		( ) Running out of propofol solution			( ) Replace solution
		The alarm sounds with the message “ELECTRODE LOOSE”:			
		( ) Failure in the transport monitor/defibrillator			( ) Correct failure, if possible ( ) Replace transport monitor/defibrillator
		Displacement/loss of nasoenteric tube:			
		The student identifies the nasoenteric tube: is externalized ( ) Yes ( ) No			( ) Inform medical team ( ) Schedule a new passage of the tube, if necessary, in the original unit
Return the patient to the intensive care unit		( ) The team returns the patient to bed			
Accommodate the patient in bed		The student switches the resources used during transport to intensive care unit resources: ( ) Transport ventilator ( ) Oxygen cylinder ( ) Transport monitor/defibrillator ( ) Saline drip stand ( ) Continuous infusion pump			
Assess the patient’s condition after returning from the transport		The student assesses: ( ) Ventilatory/respiratory conditions ( ) Hemodynamic conditions			
Record the procedure in the patient’s chart		The student records the procedure in the patient’s chart with the following information: ( ) Date ( ) Time ( ) Procedure performed ( ) Intercurrent events, if any			
The scenario must be interrupted when: at the end of the proposed activity or reaching the 10-minute time limit					
Structured debriefing			Duration: 20 minutes		
How do you feel after the simulated activity? What drew your attention the most? Why? Please mention strengths in the development of the simulated activity. Please mention points for improvement. What would you do differently? What did you learn from this simulated activity? What abilities were developed during this activity?					
References 1. Conselho Federal de Enfermagem (CFE). Resolução 376/2011. Dispõe sobre a participação da equipe de enfermagem no processo de transporte de pacientes em ambiente interno aos serviços de saúde, 2011. Brasília (DF): CFE; 2011 [citado 2017 Set 10]. Disponível em: http://www.cofen.gov.br/resoluo-cofen-n-3762011_6599.html 2. Silva R, Amante LN. Checklist para o transporte intra-hospitalar de pacientes internados na unidade de terapia intensiva. Texto Contexto Enferm. 2015;24(2):539-47. 3. Almeida AC, Neves AL, Souza CL, Garcia JH, Lopes JL, Barros AL. Intra-hospital transport of critically ill adult patients: complications related to staff, equipment and physiological factors. Acta Paul Enferm. 2012;25(3):471-6. Review. 4. Australian and New Zealand College of Anaesthetists (ANZCA). Guidelines for transport of critically ill patients. Melbourne: ANZCA; 2015 [cited 2015 Aug 30]. Available from: https://www.anzca.edu.au/resources/professional-documents/guidelines/ps52-guideline-for-transport-of-critically-ill-pat 5. Morais SA, Almeida LF. Por uma rotina no transporte intra-hospitalar: elementos fundamentais para a segurança do paciente crítico. Rev HUPE. 2013;12(3):138-46.					

RASS: Richmond Agitation and Sedation Scale; PEEP: Positive end-expiratory pressure.

## DISCUSSION

Clinical simulation has been used as a teaching strategy to come nearer to reality, in which the student effectively participates in the construction of knowledge, enabling a deeper evaluation and the acquisition of psychomotor, attitude, and cognitive skills.^(17,18)^

The simulated scenario on in-hospital transport of the critically ill patient can enable students to integrate, strengthen, and reinforce the theoretical/practical content on this subject, to assume a professional posture, and to increase confidence relative to their conduct, because of prior execution and the possibility of repetition - often impossible in clinical practice during undergraduate studies.^(19)^

One of the factors that influence the success of clinical simulation is directly related to the organization and implementation of the activities. Therefore, systematization should be considered when building the guiding instrument, taking into account the objectives to be achieved.^(1,4,17)^

Validation is fundamental in the construction of the scenario, since it verifies if it is adequate for what is proposed, enhancing credibility of the process. The use of the Delphi technique in this study provided the validation of the scenario content to be used in clinical simulation of in-hospital transport, based on a consensus among experts.^(14,20)^

This technique has the advantages of possible of access to geographically distant people, the low cost for implementation, the possibility of interaction between researcher and participants, the sharing of opinions and ideas, and the production of an instrument with high quality and specificity.^(6,7,14)^However, the Delphi technique can have disadvantages, such as the delay in returning the questionnaires, difficulties in composing the panel of experts, and requiring many rounds to establish the final consensus.^(6,7,14)^

The construction of the scenario in a structured manner makes it possible to gather a set of information, with the final objective of guiding teachers/students in the development of the simulated clinical activity. The sequence and quality of the inserted information strengthen the educational process.^(4,5,8)^

The development of the activity should include the description of a relevant topic, which works to build knowledge and promotes the recognition of priorities, planning, team interaction, effective communication, agility in decision making, and execution of the case, so that students assume responsibility for their own learning.^(1,17)^

Another important aspect of teaching through simulation is the opportunity to integrate and improve skills, besides valuing pedagogical technologies that make the teaching and learning process richer and more enjoyable, as well as favoring active learning. In the student, such techniques also awaken curiosity and the need to learn, and contribute to a more assertive attitude in face of situations experienced in the scenario.^(17-19)^With this in mind, we chose to structure the information in topics.

In the first topic, items of guidance on the theme, target audience, number of students, duration of the activity, duration of the debriefing, requirememts, general and specific objectives were available. These pieces of information are extremely important for the student to be able to develop the proposed activity. It is important to understand the level of prior knowledge of the participants, which must be compatible with the complexity of the scenario, since clinical simulation is seen as a didactic and pedagogical support technique, which provides appropriate syllabus integration and associates prior knowledge with practical experience. In addition, it favors permanent learning, according to the proposed objective and the skills to be developed in each stage of professional training.^(1)^

In the second topic, definition, relevance, importance of the theme, and the skills to be developed during care are described. Although in-hospital transport of the critically ill patient always involves a series of risks, the need for diagnostic tests, additional care, or therapeutic interventions lead to the evaluation of risks and benefits, and finally, to the indication of transporting or not the patient at a given moment. Thus, acknowledgment of the complexity of transporting a critically ill patient, and of the need for qualified professionals to assure the patient’s integrity and maintenance of life during this procedure is required.^(4,12,13)^

In the third, fourth, and fifth topics, the simulation methodology is described, with information about the clinical case, preparation of the scenario, and the necessary materials. The case description was done in an objective and structured manner for easy understanding, providing the necessary information for its development, and was based on scientific evidence, considering all steps to be developed in an authentic and real environment. Each topic was described to guide the setup of the scenario and ensure its proper use for the development of simulated best practices.

In the first scenario, the expected actions to prepare the patient to be transported were described, and in the second scenario, the expected actions to perform the transport itself. This division was suggested by the specialists so that the scenario would not take much time.

In the other items, the expected actions for each scenario are described, as well as patient, nursing care, equipment care, and communication issues, to allow the facilitator to identify what was planned and what was accomplished, and to provide immediate feedback to the students.

Debriefing is a moment that occurs right after the simulation to provide feedback and strengthen learning. It also allows the student’s free expression, through a dialogue directed towards thinking about the various areas in which they were involved, identifying the positive or negative results of the practices performed during the scene. It is essential to maintain a calm environment, so that students can share their experiences, integrating content, practices performed, and discussion of points for improvement, to gain a new understanding, consolidate learning, and practice what was learned in future events.^(21,22)^

We must also emphasize the occurrence of the most frequent adverse events during transport. They are related to hemodynamic instability, communication failure in the team, and equipment failure or lack. To provide safe care, it is necessary to improve communication among the team, standardize actions and equipment to be used during transport, and to know how to identify complications early, minimizing possible harm to the patient.^(4,9-12)^

This instrument can also be used to signal the results obtained and guide the moment of reflection during the debriefing, consolidating the construction of learning.

Clinical simulation as a teaching strategy has achieved ample space in health science education, and has contributed to greater safety in performance, as professionals. Due to its accelerated growth, this strategy deserves more studies that describe its specificities for the construction of scenarios and instruments that have a real impact on training and professional development.

The limitations of this study were the absence of a pilot test after validation and its lack of practical application with students or even professionals.

## CONCLUSION

Using the Delphi technique, in this study, we successfully constructed and validated a scenario for teaching in-hospital transport to undergraduate nursing students, which should certainly provide subsidies to reinforce the theoretical and practical content; manage emergency situations; develop preventive and corrective skills; and strengthen communication, teamwork, and clinical reasoning; besides being a tool that allows the facilitator to replicate this content in other education organizations. This scenario can be applied by several health services to evaluate the training of nursing undergraduate students, as well as for the professional improvement of those who work in the in-hospital transport service.
