# Supplementary material for: Construction and validation of an in-hospital transport simulation scenario
Source: Einstein (Sao Paulo). 2021 Sep 9;19:eAO5868. doi: 10.31744/einstein_journal/2021AO5868 (PMC8428806; doi:10.31744/einstein_journal/2021AO5868)
Supplement: Translation

# Translation: Construção e validação de cenário de simulação de transporte intra-hospitalar

**Authors:** Ellen Reis Santana, Luiz Humberto Vieri Piacezzi, Maria Carolina Barbosa Teixeira Lopes, Ruth Ester Assayag Batista, Cássia Regina Vancini-Campanharo, Aécio Flávio Teixeira de Góis

**Affiliations:** 1 Brazil Escola Paulista de Medicina, Universidade Federal de São Paulo, São Paulo, SP, Brazil.; 2 Brazil Escola Paulista de Enfermagem, Universidade Federal de São Paulo, São Paulo, SP, Brazil.

**Keywords:** Simulação, Educação em enfermagem, Transferência de pacientes

## Abstract

**Objetivo:**

Construir e validar o conteúdo de um cenário de simulação clínica para ensino do transporte intra-hospitalar de pacientes críticos.

**Métodos:**

Estudo descritivo de construção e validação de um cenário de simulação clínica para o ensino de transporte intra-hospitalar. Foi construído um cenário fundamentado na literatura seguido da validação, por meio da técnica Delphi, por cinco especialistas, com nível de concordância de 80%. Os especialistas foram selecionados por meio da amostragem do tipo bola de neve. Foi desenvolvido um instrumento contendo 26 tópicos, para serem avaliados quanto a pertinência, clareza, objetividade, exequibilidade, conteúdo atual e vocabulário. Havia ainda um campo para observações.

**Resultados:**

Foram realizadas duas rodadas pelos especialistas para a obtenção de consenso de 80%. De acordo com a recomendação dos especialistas, o cenário inicial foi escrito de forma mais clara e objetiva, e divido em duas partes: preparo do paciente e transporte do paciente.

**Conclusão:**

Neste estudo, foram realizadas, com êxito, a construção e a validação do cenário para ensino do transporte intra-hospitalar. Ele poderá ser aplicado em diversos serviços para avaliação da formação dos graduandos de enfermagem, assim como para aprimoramento profissional daqueles que atuam no serviço de transporte intra-hospitalar.

## INTRODUÇÃO

A simulação clínica é uma técnica educacional que possibilita a execução de cenários de modo a reproduzir uma situação da vida real, promover o processo de ensino e aprendizado centrado no paciente e permitir ao discente experimentar a representação de um evento sob a perspectiva clínica, além de permitir abordar grande variedade de situações em diferentes graus de complexidade, bem como o desenvolvimento de múltiplas habilidades e tomada de decisão.^(1-4)^

Com a crescente utilização da simulação clínica, como metodologia ativa no ensino das ciências da saúde, surgiu a necessidade de que fosse instituído um método para a construção de cenários, visto que a ausência de um roteiro pode comprometer a eficácia da atividade.^(4,5)^

Assim, a construção do cenário de simulação clínica exige o cumprimento de todas as etapas, incluindo levantamento bibliográfico; determinação dos objetivos; construção da situação clínica; preparação do conteúdo, do facilitador e do local e planejamento dos materiais e recursos necessários para o desenvolvimento da atividade.^(1,3)^

A descrição do cenário não deve ser fundamentada somente em experiências clínicas, pois elas podem não apresentar todas as informações necessárias para o alcance dos objetivos de aprendizagem. Nesse contexto, o processo de validação por meio de consenso entre especialistas contribui para que a simulação clínica seja efetiva, e contemple todos os itens para desenvolvimento adequado das competências a serem adquiridas.^(6-8)^

Considerando o alto índice de eventos adversos no transporte intra-hospitalar de pacientes críticos, como queda da saturação, alteração da pressão arterial e frequência cardíaca e falhas técnicas dos equipamentos utilizados, é fundamental reforçar a importância de boas práticas e realizar treinamento simulado pautado em diretrizes atualizadas, nas quais o raciocínio clínico, a avaliação otimizada e a definição dos riscos e benefícios relacionados ao transporte sejam analisados, a fim de garantir a segurança do paciente e a redução dos danos evitáveis que ameacem a vida do paciente durante o deslocamento.^(9-13)^

Desse modo, a utilização da simulação clínica para o ensino de discentes para realização do transporte intra-hospitalar de pacientes críticos torna-se de grande importância, pois permite aplicar um treinamento simulado de alta fidelidade, replicando os desafios enfrentados por profissionais da saúde no cotidiano, possíveis instabilidades e complicações clínicas, e desenvolver competências para a tomada constante de decisões complexas, prevenção de possíveis eventos adversos e estratégias que atendam às necessidades terapêuticas do paciente crítico, em um ambiente completamente seguro, controlado e, sobretudo, livre de riscos aos profissionais/pacientes.^(4,8-11)^

Considerando que a operacionalização da atividade pedagógica é relevante para o aprendizado, com a necessária associação da teoria com a prática, a utilização de estratégias que contribuam para melhor compreensão dos fenômenos é essencial para a formação dos profissionais de saúde, e, com isso, a segurança do paciente é priorizada.

## OBJETIVO

Construir e validar o conteúdo de um cenário de simulação clínica para ensino do transporte intra-hospitalar de pacientes críticos.

## MÉTODOS

Estudo descritivo de construção e validação de um cenário em atividade clínica simulada sobre transporte intra-hospitalar do paciente crítico. O estudo foi desenvolvido no período de agosto de 2017 a março de 2018, em duas etapas: na primeira, foi realizada a construção do cenário sobre transporte intra-hospitalar de pacientes críticos fundamentada na literatura. Na segunda etapa, foi realizada a validação do cenário por meio da técnica Delphi, que permite a obtenção de consenso entre um grupo de especialistas na área de conhecimento sobre determinado fenômeno – neste caso, sobre o ensino simulado na área da saúde. Quanto ao número de *experts*, não há consenso na literatura do número ideal, mas sugere-se um mínimo de cinco como suficiente para o controle de concordância.^(6,7)^Neste estudo, utilizamos cinco especialistas, e o nível de concordância considerado entre eles para validação do cenário foi de 80%, considerando que o valor recomendado na literatura é superior a 70%.^(14)^

Os especialistas foram selecionados por meio da amostragem bola de neve, e os critérios para seleção foram possuir título de Mestre e/ou Doutor na área da saúde ou educação e ter experiência mínima de 2 anos no ensino simulado na área da saúde em instituição de ensino superior.^(15)^Os especialistas receberam por e-mail a carta convite e o Termo de Consentimento Livre e Esclarecido. Após o aceite, responderam um questionário com informações para caracterização profissional e outro para avaliação o cenário.

A primeira versão do cenário foi desenvolvida com os seguintes tópicos: descrição do tema, público-alvo, pré-requisitos para participar da atividade, número de participantes, duração da cena e *debriefing*, objetivos da aprendizagem, fidelidade, complexidade, materiais necessários, orientações para preparo do cenário e ações esperadas em cada momento. Como alguns tópicos tinham mais de um item, o instrumento estruturado continha 26 itens para validação de conteúdo quanto a pertinência, clareza, objetividade, exequibilidade, conteúdo atual, vocabulário. Havia também um campo aberto para observações, caso o examinador julgasse necessário fazê-las.^(16)^

O estudo foi aprovado pelo Comitê de Ética em Pesquisa da Universidade Federal de São Paulo (CAAE: 80661017.7.0000.5505, parecer 2.503.340).

## RESULTADOS

Os especialistas selecionados para validação do cenário eram, em sua maioria, do sexo feminino (80%) e com média de idade de 43 anos (±7,33). Quanto à escolaridade, todos possuíam formação na área da saúde, e 80% possuíam título de Doutor. Em relação à experiência com simulação clínica, todos participaram do curso de capacitação para instrutor em simulação, simpósios e congressos na área, com experiência média de 9,6 anos (±6,58), sendo que 20% referiram atuar em instituições públicas, 60% em instituições privadas e 20% em ambas. Foram realizadas duas rodadas para avaliação dos especialistas para obtenção da concordância de pelo menos 80%. Na primeira, obteve-se concordância em 24 itens e discordância em dois, sendo eles a descrição do caso clínico, no qual o consenso entre os juízes foi de 40%, e no item que se referia ao tempo da atividade, no qual o consenso dos juízes foi 60% (Tabela 1).

**Tabela 1:** Discordância entre os especialistas na primeira rodada

Itens discordantes	Percentual de discordância					
	Pertinência	Clareza	Objetividade	Exequibilidade	Conteúdo atual	Vocabulário
Descrição do caso clínico (cenário)	100	40	40	100	80	80
Tempo limite da atividade (preparo para o transporte)	80	80	60	60	100	100

De acordo com a recomendação dos especialistas, o caso foi escrito de forma mais clara e objetiva, e o cenário inicial foi divido em duas partes: preparo do paciente e transporte do paciente. Cada cena teve duração de 10 minutos com *debriefing* de 20 minutos. A tabela 2 apresenta a versão final do cenário. Dessa vez, obteve-se concordância de pelo menos 80% para todos os itens avaliados.

**Tabela 2:** Versão final de cenário de simulação clínica para transporte intra-hospitalar do paciente crítico

Cenário: simulação em transporte intra-hospitalar do paciente crítico					
Tema: transporte intra-hospitalar do paciente crítico					
Público-alvo: discentes do 4º ano de enfermagem					
Pré-requisitos:	Ter realizado a aula teórica sobre transporte intra-hospitalar do paciente crítico Conhecer os recursos materiais necessários para realizar o transporte Conhecer os principais eventos adversos que ocorrem durante o transporte e como intervir				
Número de discentes: 4					
Cenário 1: preparo para transporte intra-hospilar do paciente crítico					
Atividade simulada duração: 10 minutos		*Debriefing:* 20 minutos			
Cenário 2: transporte intra-hospilar do paciente crítico					
Atividade simulada duração: 10 minutos		*Debriefing:* 20 minutos			
Ao final desse treinamento, o discente deverá ser capaz de:					
Objetivo geral:	Realizar o transporte intra-hospitalar, visando garantir a segurança e evitar/reduzir a ocorrência de eventos adversos				
Objetivos específicos:	Orientar o paciente e o acompanhante sobre a realização e a finalidade do transporte intra-hospitalar Avaliar as condições clínicas e a documentação do paciente para o transporte intra-hospitalar Planejar o transporte intra-hospitalar Reunir a equipe adequada e os equipamentos necessários para o transporte intra-hospitalar Registrar o transporte, as intercorrências e as respectivas intervenções, se houver, no prontuário				
Transporte intra-hospitalar					
Define-se transporte intra-hospitalar como a transferência temporária ou definitiva de pacientes por profissionais da saúde dentro do ambiente hospitalar, garantindo-lhes integridade e segurança^(1)^ Os pacientes críticos requerem intervenções que, muitas vezes, não podem ser realizadas no leito, necessitando de transporte para realização de procedimentos diagnósticos ou terapêuticos. Eventos adversos são frequentes durante o transporte intra-hospitalar de pacientes críticos e podem ocasionar em instabilidade respiratória e hemodinâmica e ser decorrentes, muitas vezes, de falta de conhecimento dos profissionais, falhas na comunicação entre a equipe e nos equipamentos utilizados durante o trajeto. Sendo assim, a segurança durante o transporte intra-hospitalar é de extrema importância e pode ser obtida com o ensino e o treinamento simulado de boas práticas, visando ao raciocínio clínico para avaliação precisa dos riscos e benefícios relacionados ao transporte, e ao desenvolvimento de habilidades técnicas para a sua realização, a fim de garantir a segurança do paciente e reduzir a ocorrência de danos evitáveis durante o trajeto^(2-5)^ O estudante de enfermagem precisa compreender a importância da correta identificação do paciente que será transportado, bem como da finalidade do transporte. Planejar e executar o transporte, prevenindo ou reduzindo a ocorrência de eventos adversos. Dessa forma, é fundamental que o aluno de enfermagem desenvolva as seguintes habilidades: comunicação, organização, pensamento crítico, destreza, segurança e liderança. Essas ações têm como propósito a prevenção e a redução de eventos adversos durante o transporte					
Cenário 1: preparo para transporte intra-hospitalar do paciente crítico					
*Briefing*: antes do início do cenário, realizar apresentação do caso, ambiente e recursos disponíveis para o atendimento					
Resumo do caso: Sr. J.S., de 42 anos, foi vítima de queda de aproximadamente 5m, há 48 horas e será encaminhado para o exame, pois apresentou rebaixamento do nível de consciência. Foi solicitada uma tomografia computadorizada e o Centro Diagnóstico já está aguardando. O Sr. J.S. está sedado, com curativo cefálico limpo e seco externamente, sonda nasoenteral fechada, mantendo intubação orotraqueal em ventilação mecânica – modo volume controlado, fração inspirada de oxigênio (FiO_2_): 50%, cateter venoso central em veia subclávia direita recebendo propofol a 15mL/L, e sonda vesical de demora com sistema fechado de drenagem. Não há descrição de alergias no prontuário Sinais vitais: pressão arterial (PA): 120x60mmHg; frequência cardíaca (FC): 79bpm; frequência respiratória (FR): 14rpm; saturação de oxigênio: 96% e RASS: -5					
Preparo do cenário de alta fidelidade e manequim					
Paciente/manequim deve estar no leito/cama, com o decúbito elevado, as grades elevadas e rodas travadas Paciente/manequim deve estar nas seguintes condições/com os seguintes dispositivos: sonda nasoenteral em narina direita fechada; cânula orotraqueal acoplada ao ventilador mecânico; cateter venoso central em veia subclávia direita acoplado a um sistema de infusão por bomba; pulseira de identificação em membro superior direito e sonda vesical de demora acoplada a sistema fechado de drenagem					
Materiais necessários para o cenário					
Paciente/manequim Pulseira de identificação Cama Luvas de procedimentos Cânula traqueal nº 7.0 Fixação para cânula traqueal Ventilador mecânico de transporte completo Torpedo de oxigênio Monitor/desfibrilador de transporte completo Eletrodos Atadura crepe Fita crepe Sonda nasoenteral Sonda vesical de demora Coletor fechado para sonda vesical de demora Suporte de soro acoplado à cama *Bag* de soro fisiológico 0,9% Equipo de bomba de infusão Bomba de infusão Eletrodos para monitorização cardíaca Cateter venoso central Pedido de exame Prescrição médica Folha de controle Prontuário		Maleta de transporte contendo: - Luvas de procedimentos - luvas cirúrgicas - Dispositivo bolsa-válvula-máscara - *Kit* laringoscópio com pilha reserva - Cânula traqueal (diversos tamanhos – adulto) - Fio-guia - Material para fixação da cânula - Máscara laríngea (diversos tamanhos – adulto) - Cateter venoso periférico (diversos tamanhos – adulto) - Torneirinha - Fita hipoalérgica para curativos - Seringa (diversos tamanhos) - Agulha (diversos tamanhos) - Equipo de infusão (macrogotas) - Equipo de infusão (para bomba) - Solução fisiológica 0,9% (ampolas e *bag*) - Álcool 70% - Algodão Medicamentos de emergência: - Glicose hipertônica - Adrenalina - Amiodarona - Atropina - Etomidato - Fentanil - Midazolan - Succinilcolina - Diazepam			
Preparo para o transporte					
Ações esperadas:		Orientações/parâmetros fornecidos:			
Conferir o pedido do exame e o nome completo na pulseira do paciente		( ) A identificação do pedido corresponde à identificação do paciente			
Orientar o acompanhante sobre a realização e a finalidade do transporte		( ) O aluno orienta o acompanhante sobre a necessidade de transportar o paciente para o Centro de Diagnóstico para realização de uma tomografia de crânio			
Pegar o prontuário		( ) O prontuário está na bancada			
Checar no prontuário do paciente o histórico de alergias e preparo para o exame		( ) O aluno identifica que o paciente não tem histórico de alergias e mantém jejum			
Avaliar nível de consciência/sedação do paciente		( ) O aluno identifica que o paciente está recebendo o sedativo propofol ( ) O aluno aplica a Escala de Agitação e Sedação de Richmond (RASS - *Richmond Agitation-Sedation Scale*) e identifica RASS=-5			
Avaliar a condição ventilatória/respiratória do paciente		( ) O aluno checa o posicionamento da cânula traqueal por meio da ausculta pulmonar, que está presente bilateralmente ( ) O aluno checa a fixação da cânula traqueal, que está adequada O aluno checa os parâmetros ventilatórios: ( ) Modo volume controlado ( ) Volume corrente=500mL ( ) FiO_2_=50% ( ) Frequência respiratória=14rpm ( ) PEEP=5mmHg ( ) O aluno checa a saturação de oxigênio, que está em 96%			
Avaliar a frequência cardíaca e pressão arterial		( ) O aluno avalia a frequência cardíaca, que está em 79bpm ( ) O aluno avalia a pressão arterial, que está em 120/60mmHg			
Conferir na prescrição quais infusões o paciente está recebendo e por quanto tempo		( ) O aluno identifica que o paciente está recebendo propofol em bomba de infusão a 15mL/hora e que o volume da solução (200mL) é suficiente para a realização do transporte até o retorno à unidade de terapia intensiva			
Adaptar os equipamentos necessários para o transporte		O aluno deverá adaptar: ( ) Ventilador de transporte ( ) Torpedo de oxigênio ( ) Monitor/desfibrilador de transporte ( ) Suporte de soro ( ) Bomba de infusão contínua			
Solicitar a equipe que fará o transporte		( ) O aluno solicita a equipe e a mesma está disponível			
Passagem de plantão/comunicação efetiva		A equipe que preparou o paciente comunicou a equipe que realizará o transporte intra-hospitalar, sobre: ( ) Condições clínicas do paciente ( ) Exame que será realizado ( ) Preparo para o exame			
O cenário deve ser interrompido quando: ao término da atividade proposta ou atingir o tempo limite de 10 minutos					
Cenário 2: transporte intra-hospilar do paciente crítico					
Ações esperadas:		Orientações/parâmetros fornecidos:			
Passagem de plantão/comunicação efetiva		A equipe que realizará o transporte intra-hospitalar recebeu o plantão adequadamente e confirmou: ( ) Condições clínicas do paciente ( ) Exame que será realizado ( ) Preparo para o exame			
Avaliar a distância a percorrer, possíveis obstáculos e tempo a ser despendido até o destino		( ) Os alunos discutem entre si a distância a percorrer, se haverá algum obstáculo que possa impedir o transporte e o tempo previsto			
Antecipar possíveis complicações que possam ocorrer com o paciente durante o trajeto		O aluno discute com a equipe de transporte as possíveis complicações: ( ) Deslocamento do tubo traqueal/extubação ( ) Falha no ventilador de transporte ( ) Queda de saturação ( ) Término no aporte de oxigênio do torpedo ( ) Instabilidade hemodinâmica ( ) Deslocamento/perda do acesso venoso central ( ) Término da solução de propofol ( ) Falha no monitor/desfibrilador de transporte ( ) Deslocamento/perda da sonda nasoenteral ( ) Deslocamento/perda da sonda vesical de demora			
Realizar a comunicação entre a unidade de origem e a unidade receptora do paciente		( ) O aluno comunica-se com o Centro de Diagnóstico, informando que o paciente está sendo encaminhando para a realização do exame			
Manter as grades elevadas para assegurar a integridade física do paciente		( ) O aluno eleva e mantém as grades elevadas durante todo o trajeto			
Transportar o paciente até o Centro de Diagnóstico		( ) Determinar a equipe que será responsável por realizar o transporte do paciente			
		Evento adverso		Ação esperada	
Intervir durante o transporte, caso ocorra o evento adverso		( ) Deslocamento da cânula traqueal/extubação		( ) Manter ventilação com dispositivo bolsa-válvula-máscara ( ) Auxiliar o médico a reposicionar a cânula traqueal, assim que possível	
		O paciente apresentará queda da saturação:			
		( ) Falha no ventilador de transporte			( ) Manter ventilação com dispositivo bolsa-válvula-máscara ( ) Corrigir falha, se possível ( ) Substituir ventilador de transporte
		O ventilador irá alarmar e mostrará a mensagem “BAIXO VOLUME”:			
		( ) Término no aporte de oxigênio do torpedo			( ) Substituir torpedo de oxigênio
		( ) *Cuff* da cânula traqueal desinflado			( ) Ouve o escape de ar pela boca do paciente ( ) Ausculta a região cervical do paciente e confirma a passagem de ar pelo local ( ) Verifica a baixa tensão no balonete ( ) Inflar o *cuff* até que o escape de ar seja corrigido
		Deslocamento/perda do acesso venoso central:			
		( ) aluno identifica que o acesso está exteriorizado ( ) Sim ( ) Não			( ) Puncionar novo acesso venoso periférico
		A bomba alarma com a mensagem “KVO”:			
		( ) Término da solução de propofol			( ) Substituir solução
		O aparelho alarma com a mensagem “ELETRODO SOLTO”:			
		( ) Falha no monitor/desfibrilador de transporte			( ) Corrigir falha, se possível ( ) Substituir monitor/desfibrilador de transporte
		Deslocamento/perda da sonda nasoenteral:			
		O aluno identifica que a sonda nasoenteral está exteriorizada ( ) Sim ( ) Não			( ) Comunicar equipe médica ( ) Programar nova passagem da sonda, se necessário, na unidade de origem
Retornar com o paciente para a unidade de terapia intensiva		( ) A equipe retorna com o paciente para o leito			
Acomodar o paciente no leito		O aluno troca os recursos utilizados durante o transporte para os recursos da unidade de terapia intensiva: ( ) Ventilador de transporte ( ) Torpedo de oxigênio ( ) Monitor/desfibrilador de transporte ( ) Suporte de soro ( ) Bomba de infusão contínua			
Avaliar as condições do paciente após o retorno do transporte		O aluno avalia: ( ) Condições ventilatória/respiratórias ( ) Condições hemodinâmicas			
Registrar o procedimento no prontuário do paciente		O aluno registra o procedimento no prontuário do paciente com as seguintes informações: ( ) Data ( ) Hora ( ) Procedimento realizado ( ) Intercorrências, se houver			
O cenário deve ser interrompido quando: ao término da atividade proposta ou atingir o tempo limite de 10 minutos					
*Debriefing* estruturado			Duração: 20 minutos		
Como você se sente após a realização da atividade simulada? O que mais chamou sua atenção? Por quê? Cite pontos positivos no desenvolvimento da atividade simulada Cite pontos de melhoria. O que faria diferente? O que você aprendeu com esta atividade simulada? Quais habilidades foram desenvolvidas durante esta atividade?					
Referências 1. Conselho Federal de Enfermagem (CFE). Resolução 376/2011. Dispõe sobre a participação da equipe de enfermagem no processo de transporte de pacientes em ambiente interno aos serviços de saúde, 2011. Brasília (DF): CFE; 2011 [citado 2017 Set 10]. Disponível em: http://www.cofen.gov.br/resoluo-cofen-n-3762011_6599.html 2. Silva R, Amante LN. Checklist para o transporte intra-hospitalar de pacientes internados na unidade de terapia intensiva. Texto Contexto Enferm. 2015;24(2):539-47. 3. Almeida AC, Neves AL, Souza CL, Garcia JH, Lopes JL, Barros AL. Intra-hospital transport of critically ill adult patients: complications related to staff, equipment and physiological factors. Acta Paul Enferm. 2012;25(3):471-6. Review. 4. Australian and New Zealand College of Anaesthetists (ANZCA). Guidelines for transport of critically ill patients. Melbourne: ANZCA; 2015 [cited 2015 Aug 30]. Available from: https://www.anzca.edu.au/resources/professional-documents/guidelines/ps52-guideline-for-transport-of-critically-ill-pat 5. Morais SA, Almeida LF. Por uma rotina no transporte intra-hospitalar: elementos fundamentais para a segurança do paciente crítico. Rev HUPE. 2013;12(3):138-46.					

RASS: Escala de Agitação e Sedação de Richmond; PEEP: pressão positiva expiratória final.

## DISCUSSÃO

A simulação clínica tem sido utilizada como estratégia de ensino para aproximação com a realidade, na qual o aluno participa efetivamente da construção do saber, sendo possível uma avaliação mais aprofundada e a aquisição de competências psicomotoras, atitudinais e cognitivas.^(17,18)^

O cenário simulado sobre transporte intra-hospitalar do paciente crítico pode possibilitar ao aluno integrar, fortalecer e reforçar o conteúdo teórico/prático sobre esse assunto; assumir postura profissional e aumentar a confiança em relação à sua conduta, decorrente da execução prévia e da possibilidade de repetição – muitas vezes impossível na prática clínica durante a graduação.^(19)^

Um dos fatores que influenciam no sucesso da simulação clínica está relacionado diretamente à organização e à operacionalização das atividades. Portanto, deve ser considerada a sistematização na construção do instrumento norteador, levando em conta os objetivos que se desejam alcançar.^(1,4,17)^

A validação é fundamental na construção do cenário, pois verifica se ele é adequado ao que se propõe e confere maior credibilidade ao processo. A utilização da técnica Delphi, neste estudo, proporcionou a validação do conteúdo do cenário a ser utilizado na simulação clínica em transporte intra-hospitalar, a partir de um consenso entre especialistas.^(14,20)^

Essa técnica tem como vantagens a possibilidade de acesso a pessoas geograficamente distantes, o baixo custo para operacionalização, a possibilidade de interação entre pesquisador e participantes, o compartilhamento de opiniões e ideias e a produção de um instrumento com alta qualidade e especificidade.^(6,7,14)^Entretanto, a técnica Delphi pode ter desvantagens, como demora na devolução dos questionários, dificuldades na composição do painel de especialistas e necessidade de muitas rodadas para o estabelecimento do consenso final.^(6,7,14)^

A construção do cenário de forma estruturada possibilita reunir um conjunto de informações, cujo objetivo final é nortear docentes/discentes no desenvolvimento da atividade clínica simulada. A sequência e a qualidade da informação inserida, fortalecem o processo educativo.^(4,5,8)^

O desenvolvimento da atividade deve contar com a descrição de um tema relevante, que trabalhe a construção do conhecimento e favoreça o reconhecimento de prioridades, planejamento, interação da equipe, comunicação efetiva, agilidade na tomada de decisão e execução do caso, para que o discente assuma a responsabilidade do próprio aprendizado.^(1,17)^

Outro aspecto importante do ensino por simulação é a oportunidade de integrar e aprimorar competências, além de valorizar tecnologias pedagógicas que tornam o processo de ensino e aprendizado mais rico e prazeroso, bem como favorecem uma aprendizagem ativa. Tais técnicas ainda despertam, no discente, a curiosidade e a necessidade de aprender e contribui para que as atitudes sejam mais assertivas diante de situações vivenciadas no cenário.^(17-19)^Pensando nisso, optamos por estruturar as informações em tópicos.

No primeiro tópico, foram disponibilizados itens de orientações sobre o tema, público-alvo, número de discentes, duração da atividade, duração do *debriefing*, pré-requisitos, objetivos gerais e específicos. Essas informações são de extrema relevância para que o discente consiga desenvolver a atividade proposta. Ressalta-se a importância de compreender o nível de conhecimento prévio dos participantes, que deve ser compatível com a complexidade do cenário, pois a simulação clínica é vista como uma técnica de apoio didático-pedagógica, que proporciona adequada integração curricular e associa conhecimentos prévios à experiência prática. Além de favorecer o aprendizado permanente, de acordo com o objetivo proposto e as habilidades a serem desenvolvidas em cada etapa da formação profissional.^(1)^

No segundo tópico, são descritas definição, relevância, importância do tema e as habilidades a serem desenvolvidas durante o atendimento. Embora o transporte intra-hospitalar do paciente crítico sempre envolva uma série de riscos, a necessidade de exames diagnósticos, os cuidados adicionais ou as intervenções terapêuticas levam à avaliação dos riscos e benefícios e, por fim, à indicação de transportar ou não o paciente em determinado momento. Dessa forma, demanda-se o reconhecimento da complexidade do transporte do paciente gravemente enfermo e da necessidade de profissionais qualificados para garantir a integridade do paciente, além da manutenção da vida durante esse procedimento.^(4,12,13)^

No terceiro, quarto e quinto tópicos, são descritos: a metodologia de simulação com informações sobre o caso clínico, o preparo do cenário e os materiais necessários. A descrição do caso foi realizada de forma objetiva e estruturada, para fácil compreensão, fornecendo informações necessárias para seu desenvolvimento, e esteve pautada em evidências científicas, contemplando todos os passos a serem desenvolvidos em um ambiente autêntico e real. Cada tópico foi descrito, a fim de nortear a montagem do cenário e garantir sua adequada utilização para o desenvolvimento das melhores práticas simuladas.

No primeiro cenário, foram descritas as ações esperadas para realizar o preparo do paciente que será transportado, e, no segundo cenário, as ações esperadas para realização do transporte propriamente dito. Essa divisão foi sugerida pelos especialistas para que o cenário não se prolongasse.

Nos demais itens, são descritas as ações esperadas para realização de cada cenário e questões relacionadas ao paciente, aos cuidados de enfermagem a serem prestados, aos cuidados com equipamentos e à comunicação, para permitir ao facilitador identificar o que foi planejado e o que foi realizado, a fim de fornecer um *feedback* imediato aos discentes.

O *debriefing* é um momento que ocorre logo após a simulação, para fornecer o *feedback* e fortalecer o aprendizado. Ele também permite a livre expressão do discente, por meio de um diálogo direcionado à reflexão sobre as várias áreas em que esteve envolvido, identificando os resultados positivos ou não das práticas realizadas durante a cena. É fundamental manter um ambiente calmo, para que o discente compartilhe suas experiências, integrando conteúdos, práticas realizadas e discussão dos pontos de melhoria, para obter uma nova compreensão, consolidação da aprendizagem e praticar o que foi aprendido em eventos futuros.^(21,22)^

Devemos enfatizar também a ocorrência dos eventos adversos mais frequentes durante o transporte. Eles estão diretamente relacionados à instabilidade hemodinâmica, à falha na comunicação entre a equipe e à falha ou falta de equipamentos. Para oferecer um atendimento seguro, é necessário melhorar a comunicação entre a equipe, padronizar ações e equipamentos a serem utilizados durante o transporte e saber identificar as intercorrências de forma precoce, minimizando os possíveis danos ao paciente.^(4,9-12)^

Esse instrumento pode ser também utilizado para sinalizar os resultados obtidos e nortear o momento de reflexão durante o *debriefing*, consolidando a construção do aprendizado.

A simulação clínica como estratégia de ensino tem alcançado amplo espaço no ensino de ciências da saúde e contribui para maior segurança na atuação, enquanto profissionais. Em razão de seu crescimento acelerado, essa estratégia necessita de mais estudos, que descrevam suas especificidades para construção de cenários e instrumentos que apresentem real impacto na formação e no aprimoramento profissional.

Este estudo teve como limitações a ausência de um teste-piloto após a validação e sua não aplicação na prática com os alunos ou mesmo profissionais.

## CONCLUSÃO

Neste estudo foram realizadas, com êxito, a construção e a validação, por meio da técnica Delphi, do cenário para ensino do transporte intra-hospitalar para graduandos de enfermagem, que certamente devem fornecer subsídios para reforçar o conteúdo teórico e prático; manejar situações de emergência; desenvolver habilidades preventivas e corretivas e fortalecer a comunicação, o trabalho em equipe e o raciocínio clínico, além de ser um instrumento que permite ao facilitador replicar esse conteúdo em outras instituições de ensino.

Este cenário pode ser aplicado por diversos serviços de saúde para avaliação da formação dos graduandos de enfermagem, assim como para o aprimoramento profissional daqueles que atuam no serviço de transporte intra-hospitalar.
